# Characteristics of the Aragonitic Layer in Adult Oyster Shells, *Crassostrea gigas*: Structural Study of Myostracum including the Adductor Muscle Scar

**DOI:** 10.1155/2011/742963

**Published:** 2011-06-15

**Authors:** Seung-Woo Lee, Young-Nam Jang, Jeong-Chan Kim

**Affiliations:** CO_2_ Sequestration Research Department, Korea Institute of Geoscience and Mineral Resources, Gwahangno 92, Yuseonggu, Daejeon 305-350, Republic of Korea

## Abstract

Myostracum, which is connected from the umbo to the edge of a scar, is not a single layer composed of prismatic layers, but a hierarchically complex multilayered shape composed of minerals and an organic matrix. Through the analysis of the secondary structure, the results revealed that a *β*-antiparallel structure was predominant in the mineral phase interface between the myostracum (aragonite) and bottom folia (calcite). After the complete decalcification and deproteinization, the membrane obtained from the interface between the myostracum buried in upper folia, and the bottom folia was identified as chitin. The transitional zone in the interface between the adductor muscle scar and folia are verified. The myostracum disappeared at the edge of the scar of the posterior side. From this study, the entire structure of the myostracum from the adult oyster shell of *Crassostrea gigas* could be proposed.

## 1. Introduction

The mollusk shell has been investigated as a typical biomineralization model for nearly 25 years [1–4], and it has become especially important due to its potential as a novel synthetic route to high-performance composite materials [5–8]. However, the exact understanding and a control technique on organic matrices are needed to develop a novel synthetic route [9–11]. The organic matrix intercalated in a shell is generally assumed to play a role in crystal nucleation, crystal orientation, crystal size regulation, and crystal polymorphism and to contribute to the shell's biomechanical properties [12]. Furthermore, it is reported that the organic matrix of the aragonitic layer differed from that of the calcitic layer. In mollusks with calcitic shells, one protein and one or two mucopolysaccharide bands were present, whereas in species with shells of aragonite or both aragonite and calcite, three or more proteins and three or more mucopolysaccharides were evident [13]. Additionally, the study of the interface between the aragonitic layer and calcitic layer could be a good guideline for the novel material synthesis related to organic-inorganic composites. It is reported that only two small, distinct, well-defined areas of the adult oyster shell are composed of aragonite: the myostracum and the ligament [14]. The myostracum is located in the attachment of the adductor muscle, commonly called the muscle scar or imprint, to the umbo of each valve. The adductor muscle scar (AMS) is the most conspicuous area on bivalves and *Crassostrea gigas*. The adductor muscle functions to close the shell [15, 16]. The studies on the organic matrix and the structural role of the myostracum, however, are few in number. Studies of the myostracum buried in the folia are still far from fully elucidating the structure and matrix by which it controls crystallization. Thus, the structural study of the entire myostracum is required, and the adult oyster shell of *Crassostrea gigas* has been chosen. The myostracum has significant advantages. We can study both the aragonitic layer and calcitic layer simultaneously because the matrix structure and the mechanism can be understood, and we can study the key role of the organic matrix through the physical and the chemical characteristics of the interface of the aragonitic and calcitic layers during shell formation. The information from this study could be used in the field of novel biocomposite synthesis and marine biomineral formation, such as that of bivalve shells.

## 2. Materials and Methods

### 2.1. Sample Preparation

Shells of *Crassostrea gigas* (Namhae and Tongyoung in Korea) were freshly collected, soaked in 5% NaOH, lightly scrubbed, and dried at room temperature. After they had dried completely, the samples were finely separated using a mill, cutting knife and optical microscope. The decalcified samples were prepared by submerging myostracum buried in folia into acetic acid solution. They were partly or completely removed by controlling the treatment time and the concentration of acetic acid. Deproteinization process was carried out as follows: the samples were treated by 20% sodium hydroxide at 65°C for 1 hour and then washed with distilled water. Soluble or insoluble protein obtained from the adductor muscle scar was prepared by the method of Choi and Kim [17].

### 2.2. Observation Procedure

Analysis is equipment-like as the following equipment is used to analyze a sample separated from myostracum and organic matrix. Crystal structure and crystallinity were characterized by recording a RIGAKU horizontal powder X-ray diffractometer using CuK*α* radiation via a rotating anode at 30 kV and 20 mA. Microstructures of the specimens were studied by using an Aspex personal scanning electron microscope (PSEM) with EDS capability. During SEM analysis, the features identified as organic matrix were relocated. Residual products of decalcification and deproteination were observed by scanning electron microscopy (SEM) (S-2500C HITACHI, Japan) with an acceleration voltage of 20 kV and a beam current of 5 × 10^−10^ A.

### 2.3. Amide I and Secondary Structure Determination of Proteins

The amide I feature, located approximately in the 1680–1597 cm^−1^ region, results primarily from the C=O stretching vibration coupled to the in-plane NH bending and CN stretching modes. The exact frequency of this vibration depends on the nature of hydrogen bonding involving the C=O and NH moieties; this, in turn, is determined by the particular secondary structure adopted by the polypeptide chains. The relationship between the position of the amide I band and the type of secondary structure may be best observed from the infrared spectra of homopolypeptides that adopt well-defined, and often highly homogeneous secondary structures [18, 19]. 

The spectra were recorded using a Fourier transform infrared spectrometer with a resolution of 2 cm^−1^. The system was purged with dry N_2_ to reduce interfering water vapor IR absorption and verified no water contribution by measuring KBr pellets. For intracrystalline proteins intercalated within either calcite or aragonite, the subtraction was performed [20, 21]. To obtain the undisturbed protein vibration spectra, each synthetic calcite and aragonite reference IR spectrum was subtracted from each mineral-specific layer, respectively. The subtracted spectra were analyzed by second derivatization, resolution-enhanced Fourier self-deconvolution in the amide I region and Gaussian curve-fitting using the origin 6.1. Second-derivative spectra [22] were smoothed by the 9-point FFT filter method. The number of components and their peak positions were determined by second derivatization and used as starting parameters [23]. The secondary structure contents were calculated from the area of each Gaussian band and their fraction of the total area in the amide I spectral region [24]. The assignment of these bands is based on the studies of proteins by vibrational spectroscopy.

## 3. Results and Discussion

### 3.1. Overview of the Myostracum of the Adult Oyster Shell of *C. gigas*

**Figure 1:**
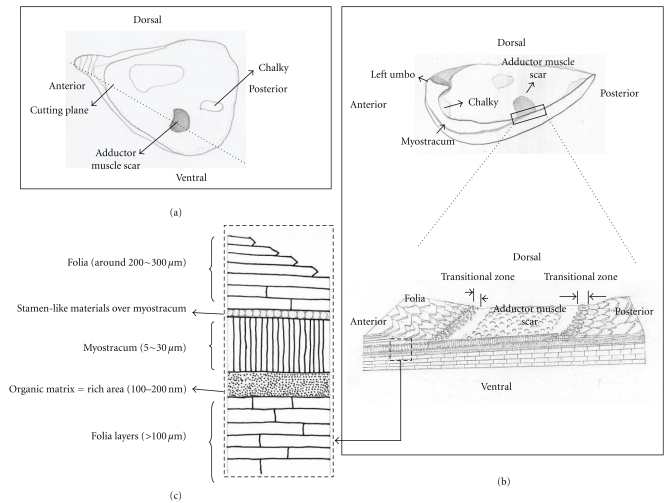
(a) Schematic of the inner surface of the left valve of an oyster shell, *Crassostrea gigas* (black dotted line: cutting plane). (b) Schematic of the vertical section with the cutting plane of the adductor muscle scar. The bottom schematic is an enlargement of the cutting plane including the AMS (adductor muscle scar), myostracum, and folia. (c) An enlargement indicating the thickness of the myostracum buried in the folia on the anterior side. The size of each layer is exaggerated for clarity. Various terms are in common use in the description of the bivalve shell [25].

The adductor muscle scar is the surface of the myostracal support for the attachment of the adductor muscle, and it is contained in the shell of most bivalves [15].  Figure 1(a) shows the inner surface of the *C. gigas* shell. Most of the area in the inner surface is composed of folia that appear to be nacreous layers and chalky layers, of which 20~30% have been identified. The folia are composed of foliated laths. The chalky layer is frequently marked by irregularly shaped, chalky-white areas and relatively soft, porous material compared to the folia [4]. The adductor muscle scar (AMS) is the most conspicuous area on the inner surface of the *C. gigas* shell. It is pigmented, and its color varies in different individuals from light, lavender-white to dark purple. The scar is located slightly in the center toward the posterodorsal side of each valve.  Figure 1(b) shows a vertical section of the center of the scar. The myostracum is elongated from the scar to the umbo. As shown in Figure 1(b), the transitional zone (approximately 0.5 mm in thickness) with granule-like material exists around the outside of the scar. Foliated laths have been developed from the anterior to the scar and from the scar to the posterior. The thickness of the myostracum in the hidden area is identified as above, approximately 10 *μ*m, but the thickness decreases to be closer to the transitional zone. Additionally, the myostracum disappears at the transitional zone (Figure 1(c)).

Figure 1(c) shows a scheme of the oyster shell with an enlargement including the myostracum. A specific stratum between the folia and myostracum could exist, but the thickness may be several hundred nanometers. Myostracum with the exception of the adductor muscle scar is buried between the foliated lath and the chalky structure. As shown in this figure, the myostracum has been surrounded by the folia; the polymorph of the myostracum is aragonite, but the folia are calcite. The shape of the myostracum is a prismatic layer (approximately 5~35 *μ*m in thickness), but that of the folia is a laminated layer (approximately 100~200 *μ*m in thickness). This single layer that comprises the myostracum is the myostracal prism, and the layer comprising the folia is the foliated lath. In this study, the interface was defined as the point of interaction between the two layers. As previously mentioned, the polymorph and the shape of the two layers are different. Thus, a buffer zone (organic matrix) could be necessary for the morphological and polymorphic control. At the organic layer, the interface between the folia and myostracum is rich at the bottom interface as compared to the upper interface. Consequently, the area including the interface between the folia and myostracum contains well-ordered arrays and structural characteristics of each layer. 

### 3.2. Characteristic Approach of the Myostracum in the Adult Muscle Scar of *C. gigas*

As previously mentioned, the point of attachment of the adduct muscle scar (AMS) is the most evident area on the interior surface of *C. gigas*. Galtsoff [25] reported that the ratio of the scar area to the shell surface area varies from 8 to 32. Although the adductor muscle is comprised of two distinct parts (approximately two-thirds of which is the anterior, translucent area, and one-third of which is the posterior, milky-white area), no microstructural differences are evident in the scar in these two areas. The study of the AMS is important because it contains the morphological and polymorphic interface. An adult oyster shell is composed of a high concentration of CaCO_3_ (above 97 wt.%) and a low concentration of organic matrix (approximately 3 wt.%) [3]. CaCO_3_ has three polymorphs, including aragonite, vaterite, and calcite. Among these, the polymorph of the myostracum is aragonite, and that of the folia is calcite. Even more interesting for this study is the hierarchical structure of the morphological interface.

**Figure 2:**
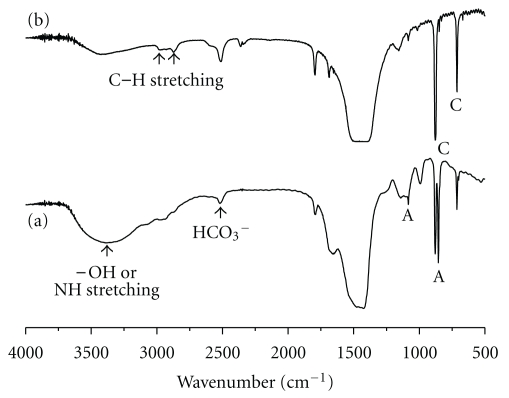
FTIR spectra of the myostracum of the AMS (adductor muscle scar) (a) and folia (b) (A: aragnonitic peak, C: calcitic peak).

From the analysis of the FTIR spectrum of the myostracum in the AMS and folia (Figure 2), the polymorph of the folia is identified as calcite, and that of the myostracum is identified as aragonite. The carbonate ions in the mineral were demonstrated by the internal vibration modes of the CO_3_^2−^ ions: 696, 713(*υ*_4_)–858, 875(*υ*_2_)–1082(*υ*_1_) and 1490(*υ*_3_) cm^−1^. The strong IR band detected at 1792 cm^−1^ could also be attributed to the C=O groups of the carbonate ions. The splitting of *υ*_4_(696), *υ*_2_(858), and *υ*_1_(1082) is characteristic of the aragonite structure. Moreover, the peak of the –OH or NH stretching (3450 cm^−1^), the CH stretching (2929 cm^−1^) as the organic matrix characteristic and the peak of HCO_3_^−^ (2650~2520 cm^−1^) that plays a role in providing the carbonate ion in Ca^2+^ protein binding in the formation of the shell are confirmed. As shown in Figure 2, organic characteristics are identified in the myostracum of the AMS and folia. Additionally, it is verified that the myostracum in the AMS has a rich organic matrix.

**Figure 3:**
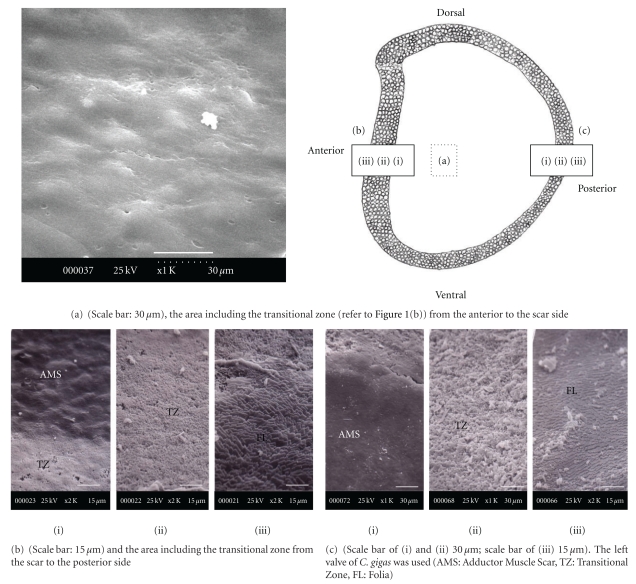
The surface image of the central adductor muscle scar.

The area between the scar and the folia contains a transitional zone with a granular-like structure, as shown in Figure 3. The surface of the central scar from which the adductor muscle has been removed with acetic acid is extremely smooth and slightly berm-like (Figure 3(a)). In the case of *C. gigas*, the ratio of the scar area in the oyster inner shell surface area is from 8 to 15. Figures 3(b) and 3(c) show the interface, or transitional zone, between the AMS and folia in both directions. The first is from the anterior side to the AMS (Figure 3(b)), and the second is from the AMS to the posterior side (Figure 3(c)). The AMS is present at the front of the posterior-advancing edge of the growing adductor muscle and shows a transitional zone of the newly deposited scar approximately 0.5 to 1 mm wide (Figures 3(b) and 3(c)). The surface of the AMS near the transitional zone typically consists of a smooth shape similar to that of the central scar. The granules between the transitional myostracum and the foliated structure on the ventral and dorsal sides of the scar are usually much narrower than that on the posterior side and are finely granular. The granule is approximately 60-*μ*m (ventral and dorsal side) to 100-*μ*m (posterior and anterior side) wide. The distinctly granular nature of this transitional zone is characteristic. The granule-to-transitional surface of *C. virginica *[15] consists of a muffin- or mulberry-like microstructure ranging in complexity from single to multiple granular mounds, in contrast to *C. gigas* in which a muffin- or mulberry-like microstructure does not exist. Moreover, Carriker et al. [15] reported that granules in *C. virginica* adhere closely between the chalky structure and plywood-like calcite. However, in the case of *C. gigas*, the granules also do not exist. From these results, a granule may be a certainly distinctive characteristic to classify the differences between *C. virginica* and *C. gigas*.

**Figure 4:**
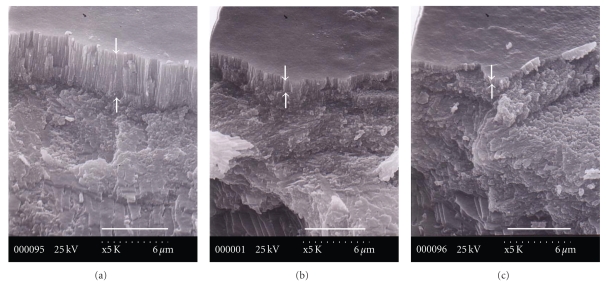
SEM images of the myostracum. (a) Central AMS (adductor muscle scar) and (b) the area between the central AMS and the edge of the scar on the posterior side (Scale bar of (a), (b), and (c) 6 *μ*m).

Figure 4 shows that the myostracum outlines in the posterior end of the scar are irregular, with reentrant angles. Especially interesting is that the thickness of the myostracal prism was rapidly tapered (indicated by the white arrows in Figure 4). As evident from Figure 4, the thickness of the myostracum decreases toward the posterior direction and then disappears in the region of the granule that exists in the interfacial posterior side. The interesting aspect is how an oyster could control a gradual decrease in the thickness. The organic matrix intercalated in the myostracum or located in the interface between the myostracum and folia could be a key factor to control the system.

Table 1 shows the difference in the amino acid composition of *C. virginica* [26] and *C. gigas *[17]. The amino acid composition of *Crassostrea gigas* was quoted in a previously published paper. As evident in Table 1, *C. gigas* and *C. virginica* have a difference in their amino acid composition according to the position and characteristics of each layer, respectively. Aspartate, serine, and glycine in *C. virginica* are rich, while serine in *C. gigas* is relatively low (Table 1). It is known that the secondary structure [27] of proteins exists mostly as a *β* structure if Asp, Gly, and Ser are mostly contained in the protein. The proportion of nonpolar residues is 0.52 in *C. virginica *and 0.47 in *C. gigas, *which is similar to Wheeler's result (0.41) [28]. The analysis by Wheeler et al. was performed on the dark and light fractions of the insoluble matrix extracted from the whole shell; therefore, a direct comparison with our data may not be appropriate. However, if their dark or light fractions are assumed to be the foliated lath and adductor muscle scar, respectively, then the data of those two reports agree relatively well (The insoluble matrix of the prismatic layer has a darker color than that of the foliated lath.) Several researchers [29, 30] reported that the amino acid composition of mollusks affected the structure of the shell and differed in the shape of the shell. A comparison between *C. gigas* and *C. virginica* in Table 1 indicates that aspartate, glycine, and serine are rich residues. Wheeler et al. reported the ratio of aspartate, glycine, and serine as 80% of the total residue.

**Figure 5:**
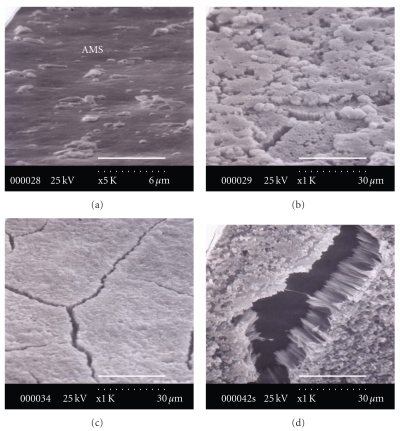
The decalcified AMS including the myostracum by acetic acid (10 wt.%) for 30 min (scale bar of (a) 6 *μ*m; scale bar of (b), (c), and (d) 30 *μ*m).

Through chemical treatment (10% acetic acid), the decalcification of myostracum in AMS was carried out (Figure 5). Granules (approximately 3 *μ*m) on the myostracum were identified (Figure 5(b)), and the decalcified myostracal prisms possessed horizontal striations along the axis (Figure 5(c)). Furthermore, the separation of the myostracum at the interface with the folia was easier to accomplish after an acidic treatment. It is estimated that the removal of the organic matrix allowed the myostracum to adhere to the folia.

### 3.3. Characteristic Approach of the Myostracum Buried in the Folia of *C. gigas*

**Figure 6:**
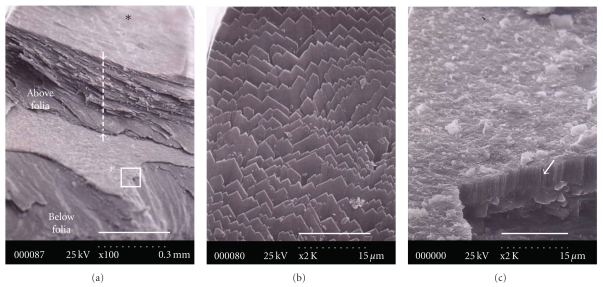
SEM images of the myostracum buried in the folia on the anterior side. (a) the area including the folia, myostracum, and folia; (b) an enlargement of the black star region; (c) an enlargement of the white star region (scale bar of (a) 0.3 mm; scale bar of (b) and (c) 15 *μ*m). The thickness of the upper folia is approximately 300 *μ*m. It appears that the myostracum was inserted in the folia.

A vertically fractured foliated lath on the anterior side shows that the thickness of the folia is from 500 *μ*m to 800 *μ*m (the white dotted line in Figure 6(a)). The folia structure does not fracture as cleanly as does the scar, but the fractures provide valuable information on the form of the laths. The surface texture ranges form smooth to slightly dimpled, parallel-lined, or chevron-marked, representing the growth halts of the crystal fronts, which point in the direction of the shell margin (Figure 6(b)). Especially in the region between the posterior side and the scar, the laths remain parallel to each other, and the growing front of each lath faces the posterior margin. The formation of the foliated laths with uniform width and length could be attributed to the influence of the mantle tissue of the hidden organic matrix. Wilbur and Saleudin (1983) reported that the shell formation occurs in the secretion by the mantle epithelial cells and in an extrapallial space in which mineral crystals are nucleated and oriented with the secreted organic matrix. The process by which the myostracum is buried in the folia could be dictated by a uniform prismatic structure into the AMS. Any organic matrix would control the structure. The most important factor is a template to control the structure and polymorphism of the myostracum.

**Figure 7:**
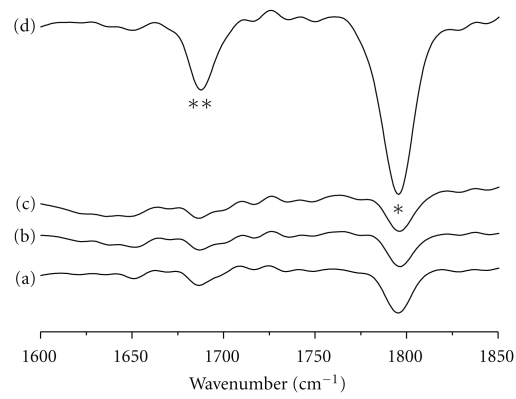
FTIR spectra of the fractured folia and myostracum on the anterior side (black star: amide I, double black stars: C=O bond). (a) Folia by fracturing in first, (b) foliated lath by fracturing in second, (c) foliated lath by fracturing in third, and (d) folia and myostracum buried in folia.

To identify the polymorphism of the myostracum buried in the folia, FTIR analysis was conducted by fracturing: the fractured outside folia (Figure 7(a)), the fractured folia lath in second (Figure 7(b)), fractured folia in third (Figure 7(c)), and the folia (Figure 7(d)) containing the myostracum. As shown in Figure 7, the intensity of the layer containing the myostracum in the amide I is higher than that in the others. The amide I feature, located approximately in the 1680–1597 cm^−1^ region, results primarily from the C=O stretching vibration coupled to the in-plane NH bending and CN stretching modes. From the above result, it may be analogized that the interface between the aragonite and calcite includes a higher content of the organic matrix than the other interfaces.

Table 2 shows that the secondary structures of the intracrystalline protein of the fractured folia (refer to Figures 7(a)–7(c)) and the layer including the myostracum (refer to Figure 7(d)) were determined by FSC and Gaussian curve fitting. When the secondary structure of the protein was analyzed by FTIR, the advantage is that the dimensional structure of the ligand in the shell tissue can be obtained without decalcification of the shell. As shown in the table, a~c (folia) mostly consist of *α*-helices, while the layer including the myostracum and folia consists mainly of *β*-antiparallel structures. The *β*-structure is a major component of the protein that forms the geometric matching needed when controlling the nuclear generation in mineralization; the *β* structure offers ligands of regular and continuous negative charge to bond with the calcium ion. It is reported that the shape of calcium carbonate that is formed in this way is determined by the protein and especially represents a conformation dependence [31]. The above information explains that the secondary structures of the protein in the folia (calcite) and myostracum (aragonite) have considerable differences. The *β*-antiparallel structure was composed a large majority in the area, which included an interface between the folia (calcite) and the myostracum (aragonite). 

From a previous research [17], it was confirmed that the organic matrix faced the crystal (001) plane of the myostracum of the AMS and folia. The structural or geometric matching at the inorganic-organic interfaces is a key concept in oriented nucleation in biomineralization. Mann [31] explained the specific nucleation of the (001) face of the aragonite on the surface of highly acidic *β*-anti proteins in the shell nacre. The results show that the a- and b-axes of the *β*-anti of the matrix are aligned with the a- and b-crystallographic directions of the aragonite lattice. That is, the crystals are oriented such that the (001) crystal face (the a-b plane) and c axis are parallel and perpendicular to the underlying matrix surface, respectively. Thus, the crystal orientation (001) of the myostracum composed of aragonite could indicate the identity of the *β*-structure in the myostracum buried in the folia. This result, moreover, incorporates both an ionotropic and an epitaxial formation of the myostracum that could be induced by the *β*-structure. Of course, only the *β*-structure is a major factor in accounting for the formation of the myostracum and the control of the interface between the myostracum and folia.

**Figure 8:**
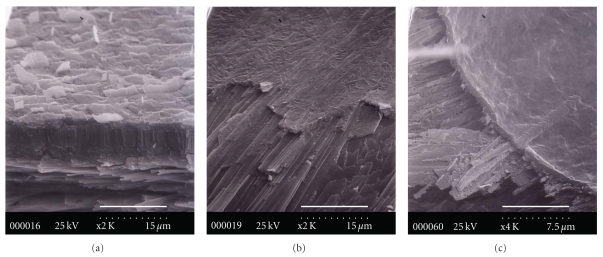
The decalcified myostracum buried in folia by acetic acid (10 wt.%). (a) fractured myostracum including the folia; (b) decalcification for 30 min; (c) decalcification for 1 hr (scale bar of (a) and (b) 15 *μ*m; scale bar of (c) 7.5 *μ*m).

The optical microscope is used to verify the separation of the myostracum at the boundary of the folia after fracturing.  Figure 8 shows the results; within 30 min of decalcification, the myostracal prism is verified (Figure 8(a)), and lawn-like material is also verified (Figure 8(b)). After the myostracal prism is eliminated, a membrane is verified (Figure 8(c)). The morphology and shape of the membrane differ from previous minerals. It can be analogized that the organic matrix in contact with the myostracum is mostly soluble, while the membrane to be eliminated from the myostracal prism was occupied by a major portion of insoluble matrix. It is known that proteins extracted from the shell play an important role in biomineralization. Numerous experiments grown *in vitro *in crystals of proteins extracted from the shell have been performed to elucidate their functions during the formation of either aragonite or calcite. From various *in vitro* experiments, some researchers [32] reported that a polyanionic-soluble protein determines the phase of the calcium carbonate, while other researchers have reported that an insoluble protein determines the phase [33]. Belcher et al. [34] also produced an *in vitro* system capable of specifically inducing the formation of aragonite and calcite crystals in the presence of appropriate acidic macromolecules extracted from nacre. This specificity is dependent not only on the acidic macromolecules but also requires the presence of *β*-chitin, as opposed to *α*-chitin, and silk fibroin [35]; chitin has an intimate relationship among proteins of the silk-fiber type, rich in Gly and Ala [36]. As shown in Table 1, Gly in *C. gigas* and *C. virginica* is rich (27~35%), and Ala of the insoluble portion of *C. gigas* is approximately three times higher than that of *C. virginica*. The interface between the myostracum, and folia may have a significant effect on the mechanical properties of the overall composites.

**Figure 9:**
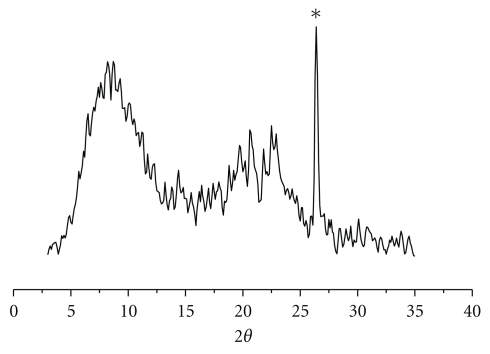
XRD-diffraction profile of the membrane obtained from the myostracum buried in folia (* indicates the main peak of aragonite).

After the complete decalcification, the membrane obtained from the interface of the myostracum buried in the folia was deproteinized. After eliminating the insoluble protein, the membrane was identified by XRD analysis (Figure 9); it is verified that the organic membrane from the myostracum buried in the folia possesses chitin-like characteristics. It is known that the peaks of *β*-chitin are 9.2 and 20.3 for the 2*θ* value. In the case of the mambrane, both peaks, a strong peak at 9.2 and a weak peak at 20.3, were identified. Above all, *β*-chitin has a good relationship with the *β*-structure of the protein secondary structure for the nacre formation [37]. Thus, the correlation between the organic matrix including the *β*-structure and chitin and the formation of the myostracum buried in the folia could be inferred. 

The study on the interface with different polymorph and morphology is significant for the synthesis of hierarchical composites with organic-inorganic interactions. The information obtained from this study will be valuable for marine biomineralogists and material scientists.

## 4. Conclusions

This study presents a comprehensive characterization of the structure, minerals, and organic matrix of the interface between aragonite and calcite in *Crassostrea gigas*.

- The myostracum from the umbo to the scar is a hierarchically complex multilayer composed of a myostracal prism and organic matrix. The thickness of the myostracum was rapidly tapered at the end of the posterior side of the scar.
- Although *Crassostrea virginica *and* Crassostrea gigas* belong to the same genus and are similar in shape, there are differences between them, including the Quenstedt muscle scar, granules buried in the foliated lath, and the lack of a muffin-like microstructure.
- The myostracum was identified as a prismatic layer oriented to (001). A *β*-antiparallel structure was predominant at the interface between the myostracum (aragonite) and the bottom folia (calcite). Thus, the *β*-antiparallel structure may be attributed to the orientation of the myostracal prism.
- After the complete decalcification and proteinization, the membrane obtained from the interface of the myostracum buried in the folia was identified by XRD analysis; it was verified that the organic matrix is characteristic of chitin.

The myostracum of the oyster shell of *C. gigas* is a hierarchical structure from the cooperation between the organic matrices (protein and polysaccharide). The polymorph and the morphology have been controlled by their close interaction.

## Figures and Tables

**Table 1:** Amino acid compositions of the soluble and insoluble proteins from *Crassostrea virginica* [26] and *Crassostrea gigas* [17] (Asx: Asp + Asn, Glx: Glu + Gln).

Species	Layer	Asx	Thr	Ser	Glx	Pro	Gly	Ala	Val	Tyr
*C. virginica*	Folia	29.4	0.8	22.0	5.2	3.5	33.4	1.42	1.2	0
	Whole shell, insoluble only	18.1	1.2	14.2	3.5	6.2	34.1	4.7	3.0	4.9

*C. gigas*	Folia	24.8	1.9	6.6	4.2	5.5	29.3	10.3	3.0	2.2
	Whole shell, insoluble only	8.7	2.8	5.8	6.3	6.9	27.4	12.4	5.6	2.0
	Myostracum in AMS	20.9	2.7	6.4	7.6	5.1	30.0	6.5	3.2	1.8

**Table 2:** Secondary structure of the intracrystalline proteins of each layer *in vivo*: (a) Folia by fracturing in first, (b) Folia by fracturing in second, (c) Folia by fracturing in third, and (d) Folia and myostracum buried in folia.

Assignment	Band position (cm^−1^)	(a)	(b)	(c)	(d)
*α*-helix	1647–1660	58.0	58.5	66.1	8.6
*β*-sheet	1615–1640	7.7	9.8	9.0	13.1
Turns	1661–1680	—	—	3.9	3.8
*β*-anti	1681–1692	34.3	31.7	21.0	74.5

Unit: area %.
